# Vegetation dynamics at the upper elevational limit of vascular plants in Himalaya

**DOI:** 10.1038/srep24881

**Published:** 2016-05-04

**Authors:** Jiri Dolezal, Miroslav Dvorsky, Martin Kopecky, Pierre Liancourt, Inga Hiiesalu, Martin Macek, Jan Altman, Zuzana Chlumska, Klara Rehakova, Katerina Capkova, Jakub Borovec, Ondrej Mudrak, Jan Wild, Fritz Schweingruber

**Affiliations:** 1 Institute of Botany, The Czech Academy of Sciences, Zamek 1, 252 43, Pruhonice, Czech Republic; 2 Department of Botany, Faculty of Science, University of South Bohemia, Na Zlate stoce 1, 370 05, Ceske Budejovice, Czech Republic; 3 Biology Centre, The Czech Academy of Sciences, Branisovska 31, 370 05 Ceske Budejovice, Czech Republic; 4 Swiss Federal Research Institute WSL, Birmensdorf, Switzerland

## Abstract

A rapid warming in Himalayas is predicted to increase plant upper distributional limits, vegetation cover and abundance of species adapted to warmer climate. We explored these predictions in NW Himalayas, by revisiting uppermost plant populations after ten years (2003–2013), detailed monitoring of vegetation changes in permanent plots (2009–2012), and age analysis of plants growing from 5500 to 6150 m. Plant traits and microclimate variables were recorded to explain observed vegetation changes. The elevation limits of several species shifted up to 6150 m, about 150 vertical meters above the limit of continuous plant distribution. The plant age analysis corroborated the hypothesis of warming-driven uphill migration. However, the impact of warming interacts with increasing precipitation and physical disturbance. The extreme summer snowfall event in 2010 is likely responsible for substantial decrease in plant cover in both alpine and subnival vegetation and compositional shift towards species preferring wetter habitats. Simultaneous increase in summer temperature and precipitation caused rapid snow melt and, coupled with frequent night frosts, generated multiple freeze-thaw cycles detrimental to subnival plants. Our results suggest that plant species responses to ongoing climate change will not be unidirectional upward range shifts but rather multi-dimensional, species-specific and spatially variable.

Cold biomes at high latitudes or high elevations are among the most dynamic systems on earth due to rising temperatures^1^,^2^. Arcto-alpine biomes warm faster than global average^2^, leading to increase in relative abundance of species adapted to higher temperature^3^, a process known as thermophilization^4^. Consequently, species which are narrowly specialized to cold habitats migrate upwards or experience local extinctions^5^. Ongoing distribution shifts^6^,^7^ and changes in plant cover, including the colonization of newly deglaciated areas^8^ are widely acknowledged^9^. Rapid warming is also changing plant phenology^10^, growth^11^ and productivity^12^.

Most of our knowledge about the recent vegetation changes in arcto-alpine systems comes from humid regions^5^,^7^,^13^,^14^,^15^. While ongoing warming is likely to be the key driver of vegetation change in these cold and humid regions^3^, altered precipitation regime and its subsequent effect on soil water balance may be even more important in dry regions^16^,^17^,^18^. Changes in precipitation regime are often accompanied by an increase in extreme events^2^ to which arid regions seem to be more vulnerable^19^. For instance, extreme precipitation in arid mountains can cause floods and landslides on poorly vegetated slopes^20^ and the increased soil moisture together with associated frost heave can uproot plants^21^. Moreover, plant-plant interactions are likely to differ between humid and arid ecosystems^22^,^23^. Because of the specific precipitation regime, arid regions may not undergo the processes of thermophilization generally described in cold biomes^3^,^5^. The lack of knowledge about vegetation dynamics in response to climate change in cold and dry biomes is striking especially in dry continental Asia, where vascular plants grow in the highest elevations on earth.

Ladakh, an arid mountainous region in the NW Himalayas, represents a unique region for studying vegetation dynamics and plant responses to climate change (Fig. 1 and Supplementary Fig. S1). Due to the dry continental climate, the mountains of Ladakh are often unglaciated up to 6200–6400 m. The unglaciated terrain together with dry climate allows plant species to growth in extreme elevation even above 6000 m^24^. Still, a relatively large and potentially colonizable area is found above the current upper distributional limit of vascular plants within this region. The mean temperatures in Ladakh have increased by 1.7 °C during the last century (Supplementary Fig. S2), and the warming accelerated in the 1990s^25^ leading to rapid glacier retreat^26^. Beside warming, Ladakh also experienced increasing precipitation, accompanied by more frequent extreme snowfall and storm events causing severe floods^27^,^28^,^29^.

Plant responses to environmental changes, including climate warming and increased precipitation, can be predicted from their functional traits^30^. Plant tissue nutrient content is one of the indicators of plant growth limitation at high elevations^31^,^32^. High nutrient but also carbohydrate concentrations commonly found in alpine plants indicate the plant’s inability to use the absorbed resources for growth (sink limitation) due to low temperatures restriction on enzymatic processes. Climate warming in the cold NW Himalayas can reduce sink limitation and increase relative abundance of faster growing taller species with optima at lower elevation steppes and semi-deserts. Contrary, increased precipitation and soil disturbance can support species from wet habitats such as springs and snowbeds, which are better adapted to repeated soil freezing and thawing. Hence, altered precipitation regime in the alpine and subnival zones of dry Himalayas can lead to the process of hygrophilization rather than thermophilization.

To explore upward plant migration and susceptibility of the cold and dry region of Ladakh to climate change, we (1) revisit an area extensively surveyed 10 years ago to find possible outpost populations above the previously recorded altitudinal upper limit; (2) re-survey permanent plots established in the alpine and the subnival belt after 4 years characterized by unprecedented warming and extreme summer precipitation; (3) characterize plant morphological and ecophysiological adaptation to evaluate the biological mechanisms of vegetation changes; (4) perform anatomical determination of plant age and growth rate. Plant age provides crucial proof of recent colonization, and recent advances in anatomical assessment of distinct annual rings enables age determination, even in plants with a root collar thinner than 1 mm^33^. Plants from cold unproductive environments tend to invest into longevity at the expense of fast growth, thus older plants are usually found at higher elevations^34^. However, warming alleviates abiotic stress and reduces the need to invest into longevity, which in turn can trigger uphill plant migration^4^,^35^,^36^. If this assumption is true, we can expect a negative relationship between elevation and plant age at the higher end of the elevational gradient. We hypothesize that the ongoing climate warming has promoted uphill plant migration in this cold and dry region, so that absolute upper limits, species richness and cover of vascular plants increased in the past decade. If so, the species increasing in cover in the subnival belt should have syndromes of traits characteristic for lower elevation habitats. On the other hand, the evidences of thermophilization might be overridden by increased frequency of extreme snowfall events producing additional disturbance resulting in reduced species richness and cover.

## Results

### Climate changes

Overall, the period studied was characterized by warmer temperatures and extreme snowfall events (Supplementary Fig. S2). Long-term meteorological (1901–2014) records of temperature and precipitation in Ladakh show positive trends in mean winter, spring and fall temperature, with pronounced warming since the mid 1990s. The records also show a substantial increase in total precipitation and summer temperature since the mid 1990s. *In-situ* climate records from our study area for the period 2008–2014 confirm the general trend. There was a significant increase in mean daily maximum T for June (increase of 2.43 °C per year), July (+1.42 °C per year), August (+0.95 °C per year), and September (+1.02 °C per year; Fig. 2B and Supplementary Table S1). Similar trends were recorded for daily mean T in the summer months, expect for August (Fig. 2A). The mean summer T (June to August) increased by 0.59 °C per year. The diurnal T range also increased (Fig. 2C). No major changes were recorded for winter month T, except for a cooling in February (−0.38 °C per year). The growing season length increased from 56 days in 2009 to 83 in 2010, 79 in 2012, and 75 in 2013. The summer 2010 was snowiest in past eight years (Fig. 2D).

### Plant distributional limits and their upward shifts

During the first survey in 2001–2003, nine species reached 6000 m and above it: *Aphragmus oxycarpus*, *Draba altaica* and *Draba oreades* were found highest at 6000 m, *Ladakiella klimesii, Stellaria decumbens* and *Saussurea glacialis* at 6010 m, *Poa attenuata* and *Waldheimia tridactylites* at 6030 m, and *Saussurea inversa* at 6060 m (Supplementary Figs S1 and S6). During the second survey in 2010–2013, there was one new species extending its range above 6000 m (*Saussurea gnaphalodes*). In total, the five species were found at higher elevations, the highest one being at 6150 m. These shifts extended the vertical range by 140 m in *Ladakiella klimesii*, 150 m in *Poa attenuata* and *Draba altaica*, 120 m in *Waldheimia tridactylites,* and 180 m in *Saussurea gnaphalodes.*

### Changes in diversity and composition in permanent plots

Total plant cover decreased both in the alpine (GLMM: chi-square = 94.4, P < 0.001,) and subnival (GLMM: chi-square = 35.7, P < 0.001, Fig. 3) vegetation between 2009 and 2012.

Species richness showed no significant change in alpine vegetation (GLMM: chi-square = 3.607, P = 0.998, Supplementary Fig. S7), and marginally decreased in the subnival vegetation (GLMM: chi-square = 3.607, P = 0.067). The species composition changed between resurveys in both the alpine (standardized RDA, F = 0.2, P = 0.002, Supplementary Fig. S8) and subnival (the only significant statistic was non-standardized RDA, F = 1.4, P = 0.008) vegetation. In the subnival belt this was mostly due to changes in abundance and not floristic turnover.

The mean total cover per plot decreased from 46 to 31% in the alpine (mean relative change 35%), and from 17 to 10% (mean relative change 48%) in the subnival zone between the two sampling periods. 20 species (6 alpine and 14 subnival, Supplementary Table S2) significantly decreased in plant cover (mean relative change 40.1%), no species significantly increased in their cover. From 41 species recorded during the 2009 survey, three species were not found, and five new species appeared during the 2012 survey; all of these losses and gains happened within the alpine vegetation.

### Plant age distribution along elevational gradient

The alpine belt hosts species with a higher mean age while plants are usually younger and grow slower in the subnival belt (Fig. 4). The mean age of alpine and subnival plants was 20.5 and 11.4 years, respectively. The oldest plant individuals were *Potentilla pamirica* (70 years) and *Arenaria bryophylla* (53 years) in the alpine zone at around 5700 m.

**Figure 1:**
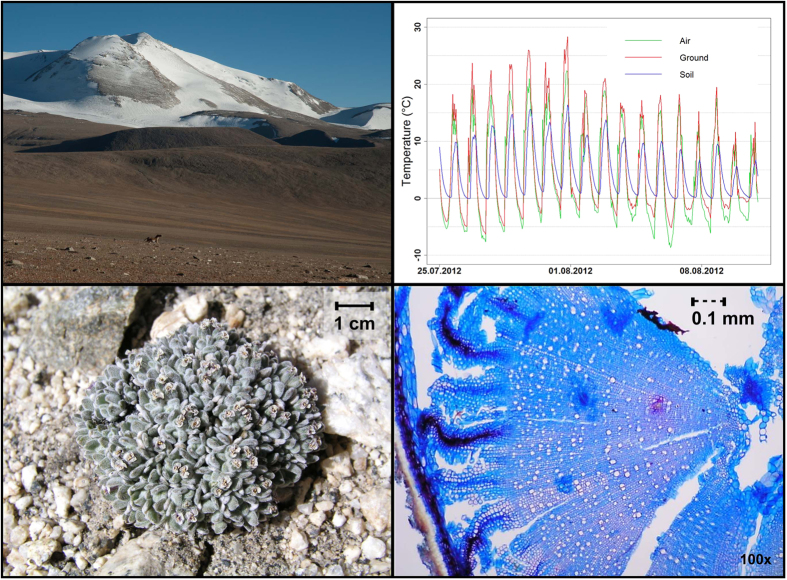
Study area in Eastern Ladakh, NW Himalayas. (A) Subnival belt around Chamser Kangri peak (6660 m a.s.l.). (B) Daily course of air, surface and soil temperatures in the peak of the vegetation season at 6150 m; the key for the survival of plants here is the rooting zone temperature during the growing season. From July to August, plants are able to withstand freezing air temperatures almost every night for 5–10 hours, but they never occur where the rooting zone temperature falls deeply below zero during the period of active growth. (C) The oldest plant (22 years) at 6150 m was an endemic species *Ladakiella klimesii*. (D) Its age was derived from ring counts on a 0.7 mm radius of root collar.

The most individuals found above 6000 m were established within the last decade (e.g. *D. altaica*, one specimen 8 year-old; *W. tridactylites*, three specimens 4, 4 and 9 years-old; *S. inversa,* three specimens 2, 6 and 6 years-old). The oldest plant individual at the highest site 6150 m was a 22 year-old endemic *L. klimesii* (Fig. 1D), and the two other specimens of this species were 8- and 16 years-old, indicating that some of outpost populations above 6000 m were established at least two decades ago.

### Plant functional traits

Plant trait values differ significantly between alpine and subnival vegetation (Fig. 5, Table 1). 54% of the alpine species are clonal species with adventitious roots (spreading splitters) whereas subnival vegetation is dominated by small non-clonal cushion species (64% of the subnival species) with a main tap root producing belowground branches bearing aboveground leaf rosettes (spreading integrators). Subnival species are in average 21% shorter, 44% younger, grow 25% slower, have 48% lower total dry biomass and 36.9% smaller rooting depth than alpine species (Table 1, Supplementary Fig. S9). Subnival species have significantly higher nitrogen concentration in their leaves (6.9%) and roots (30.6% respectively) as well as higher phosphorus concentration in roots (27.9%), but lower LDMC (25.3%), leaf carbon content (2.9%) and consequently lower leaf C:N ratio (8.6%) compared to alpine species. In addition, subnival species have 9.2% more soluble carbohydrates and 76.1% more fructans in their roots but do not differ in starch content compared to alpine species. Finally, subnival communities are composed of plant species with an affinity for lower soil moisture and soil salinity compared to species from alpine communities (Table 1).

### Shift in plant strategies in response to changing environmental conditions

Compositional changes observed in alpine and subnival vegetation from 2009 to 2012 were not ecologically random as evidenced from a shift in community-weighted mean trait values (Table 1). These changes in the CWM correspond to the decrease in abundance of the species with syndromes of highest elevation described in the previous section. The proportion of non-clonal species with a main tap root decreased between sampling periods (5.9% in alpine and 10.1% in subnival habitats), while that of clonal plants with adventitious roots increased (4.9 and 17.5%, respectively). This was accompanied by a significant decrease in root nitrogen (2.7 and 3.9%) and phosphorus (3.5 and 3.6%) concentrations. There was also a significant decrease in mean seed weight (7.1%) and increase in LDMC (4.4%) in alpine vegetation and increase in plant height (9.6%) in the subnival zone. Indicator values for soil moisture and salinity changed significantly between sampling periods in the alpine vegetation: species with an affinity towards wet and saline habitats (e.g. *Potentilla gelida*, *Halerpestes sarmentosa*, *Pegaeophyton scapiflorum*, *Koenigia islandica*) increased in cover relative to species from dry habitats (e.g. *D. altaica*, *P. pamirica*, *S. gnaphalodes*, *D. oreades*, Supplementary Fig. S8). In subnival vegetation, species with optima at higher elevations decreased in cover.

An alternative species-based approach confirmed that soil moisture indicator value is the best predictor of changes in species’ cover in the alpine vegetation (Fig. 6). Species preferring drier surfaces were most prone to decline in their cover. The cover values of this group was further predicted by leaf δ^13^C as a proxy of WUE, root phosphorus concentration as a proxy of growth rate, and surface stability as a proxy of disturbance. Species preferring drier and stable surfaces with lower δ^13^C and higher RPC were more susceptible to decline in cover than species with higher δ^13^C and faster growth preferring wetter and naturally more disturbed habitats. In the subnival vegetation the only significant trait predicting changes in their abundance was space occupancy strategy (Supplementary Fig. S10). Species capable of clonal multiplication belonging to category of spreading splitters (*Carex sagaensis*) and spreading integrators (*Oxytropis platysema*, *W. tridactylites*, *Desideria pumila*) increased in cover, while non-clonal species with main tap roots belonging to category of non-spreading intergrators (*A. oxycarpus*, *D. oreades*, *S. hypsipeta*, *L. klimesii*) or species with limited vegetative reproduction belonging to category of non-spreading splitters (*P. attenuata*, *Festuca non-coelestis*) were more prone to decrease in cover.

## Discussion

The combination of site re-visitation, age determination and trait characterization did not provide evidence for thermophilization at the world upper limit for vascular plant life after an exceptionally warm decade. However, our results from the arid Himalayas do indicate the sensitivity of vegetation to the ongoing climate change. Unlike in the humid and cold high-altitude regions^3^,^5^, the combination of warming and increased frequency of extreme snowfall events in the arid Himalayas^27^ causes increasing disturbance events rather than purely ameliorating the level of stress. This results in a more complex pattern of vegetation changes than otherwise expected by the upward migration and thermofilization hypotheses^7^,^37^.

### Plant uphill migration and adaptive strategies

Accelerated warming in Eastern Ladakh over the past decade may have promoted the establishment of new populations at very high elevation, similarly as in other mountains^5^,^13^,^14^,^38^. We discovered several outpost populations during the re-exploration of the Tso Moriri area above the upper vegetation limit determined 10 years before this study. While plant age analysis indicates that the highest outpost of *L. klimesii* was overlooked in the previous survey, *L. klimesii* was the only plant species of the highest outpost that was over 10 years old. The other species have established within the warmer last decade. Also the younger age of the subnival species compared to the alpine species indicates recent establishments which coincide with the recent accelerated warming.

The observed upward plant migration was not ecologically random process as evidenced from plant life-history analyses^37^. Subnival species that significantly extended their range by uphill shift evolved similar anatomical and ecophysiological adaptations despite differences in geographical origin, vertical distribution, thermal optima and phylogeny^24^. Common features to all of them are the absence of fibers and the presence of thick-walled, and small and short vessels (10–30 μm in diameter and 40–150 μm in length) which protect the plants against drought- and frost-induced cavitation and embolism^39^. They are cushion-forming perennials with secondary growth, annual rings of semi-ring porosity and very small annual increments (ring width 0.03–0.05 mm) reflecting the harsh environmental conditions. These subnival species contain more nutrients and soluble carbohydrates and are capable of maximizing the rate of photosynthesis during short periods of favorable conditions, due to high foliar nitrogen content^31^ (Table 1 and Supplementary Fig. S9). Higher concentrations of simple sugars and sugar alcohols are thought to serve as osmoprotectants when drought or frost cause water deficit in the plant^40^. These adaptations are crucial, because drought on sunny days and frost in cold nights are very common. Beside proper ecophysiological adaptations, the migrating species are anemochorous^37^, such as Saussurea spp. from the Asteraceae family, which produces a large quantity of small wind-dispersed seeds with an attached feathery pappus, or Draba spp. and *A. oxycarpus* from the Brassicaceae family which have extremely light seeds^24^.

While proper organismal adaptations to freezing temperatures and drought are necessary prerequisite for upward plant migration^41^, it remains question if there are some shared physiological constraints beyond which plants cannot move^31^. There is increasing evidence that the coldest places on Earth with angiosperm plant life are determined by a growing season (defined by a daily mean >0 °C in the rooting zone) of at least 50 days, with seasonal mean root zone temperature around 2–3 °C^32^. This could be considered as the universal minimum for persistent angiosperm life^32^, although further evidence is needed. Klimes and Dolezal^42^ found the upper limit of plant life at 6030 m on the western slope of the Chalung mountains in 2002 (10 km north of our study site), formed by *Poa attenuata* and *Waldheimia tridactylites*; in-situ measurements over 2002–2003 showed a 47 day growing season with mean root zone (−5 cm) T of only 1.7 °C. Unfortunately, the existence of plant life at this precisely georeferenced site was not confirmed during the revisitation in August 2013 (Dolezal, unpublished data), indicating the transient character of outpost populations in extreme elevations. Interestingly, what is common for the uppermost plant populations for which in-situ measured climate data are available (Mt Chalung 6030 m and Mt Shukule 6150 m), despite the difference in absolute elevations and seasonal mean T (1.7 °C vs 2.8 °C), is at least four continuous weeks without frost in the rooting zone. This suggests that the temperature of the rooting zone during the growing season is more important for vascular plant life at its upper limit than other commonly used climate measures. The four-year records from 6150 m indicate that freezing of the soil is more critical for vascular plants than frost above the ground^31^,^32^. While the air temperature during the short growing season drops below zero every night for 5–10 hours, repeatedly to about −5 °C (Supplementary Fig. S11), the soil temperature remains above zero (Fig. 1B). Plants more easily cope with freezing air than soil because of the differences between shoot epidermis and root rhizodermis; the root rhizodermis represents a less sufficient barrier against ice-nucleation upon contact with extrinsic soil ice masses, especially in juvenile plants^43^. Most of the upper subnival species in Ladakh have a thick bark, large cortex and a xylem with much living parenchyma with high soluble carbohydrate concentrations (Fig. 1 and Supplementary Fig. S9) as protection against night frost during the short summer season.

### Thermophilization or hygrophilization?

In addition to increasing temperatures during the past decade, the other aspect of climate change in the region, the extreme snow fall events^27^ and the subsequent frost heave (Supplementary Fig. S5), when the soil is saturated with water, could pose a serious challenge to vascular plants adapted to arid soils of cold alpine deserts^31^,^43^. The extreme snow fall events during the study period coincide with the decrease in vegetation cover (increase in bare ground) by roughly one third, for both alpine and subnival communities. Such short term climate events can have long-lasting effects^44^. While increased temperature could promote plant regeneration, the very slow growth rate common to species in this system, is unlikely to compensate for the increased frequency and intensity of the disturbance.

Trait analyses of the re-surveyed alpine and subnival vegetation did not provide support for thermophilization processes. Evidence for thermophilization would have been indicated by an increase in the abundance of species with trait syndromes and elevational optima common for thermophilous or xeric species from habitats of lower elevations, such as steppes and deserts^45^. Instead, the vegetation composition in permanent plots in the alpine belt shifted towards clonal species preferring wetter habitats (Table 1). We observed an increase in hygrophytes, a phylogenetically diverse group of plants growing in permanently or temporarily wet soils that are sometimes waterlogged and frozen almost every night on the surface during summer. These were not only species with high affinity to soil moisture, but also those with higher leaf δC^13^ (higher WUE) and lower P concentration. This indicates that increasing moisture is not the only cause of the observed vegetation change but also increased disturbance due to repeated freeze-thaw cycles, which plants with higher WUE can better cope with. The higher P content is characteristic of slow growing alpine specialists^24^,^31^,^32^. Most prone to decline are therefore species with lower WUE and slow growth not capable of clonal multiplication occupying drier and stable surfaces, i.e. the common species at the highest elevations in arid NW Himalayas.

**Figure 2:**
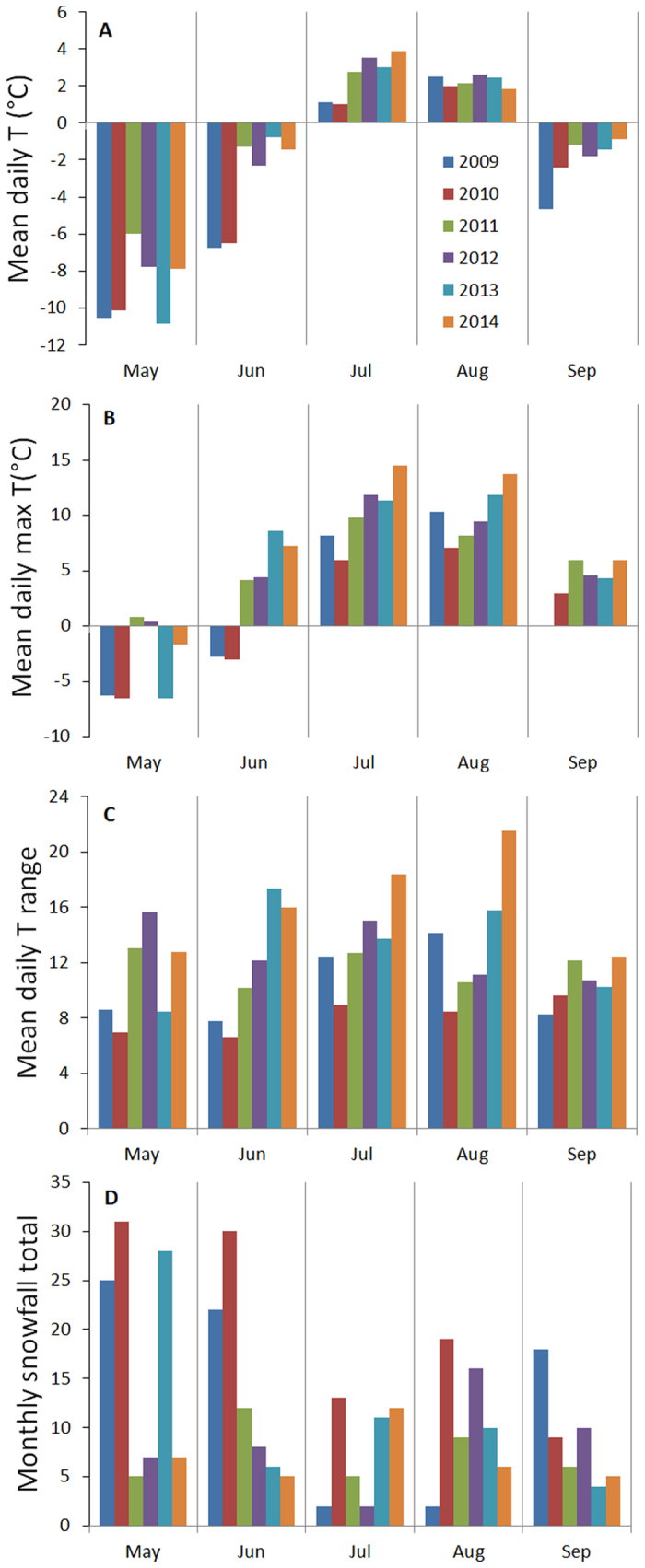
Climate conditions at the study area between January 2009 and September 2014. Mean and maximum monthly temperatures (T), the number of days with snow cover (snowfall total) and difference betweem maximum and minimum daily T (range) are shown.

**Figure 3:**
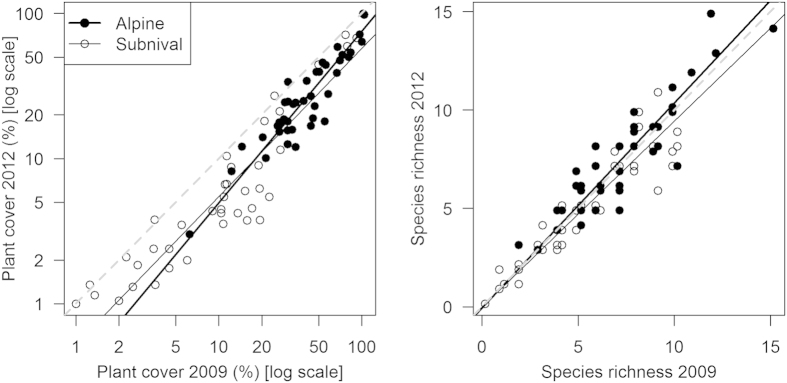
Changes in plant cover and species richness in the alpine and subnival vegetation between August 2009 and 2012. The dashed line represents the null hypothesis of no change in cover or richness between surveys, and the solid lines represent a linear regression fitted to the alpine and subnival data.

**Figure 4:**
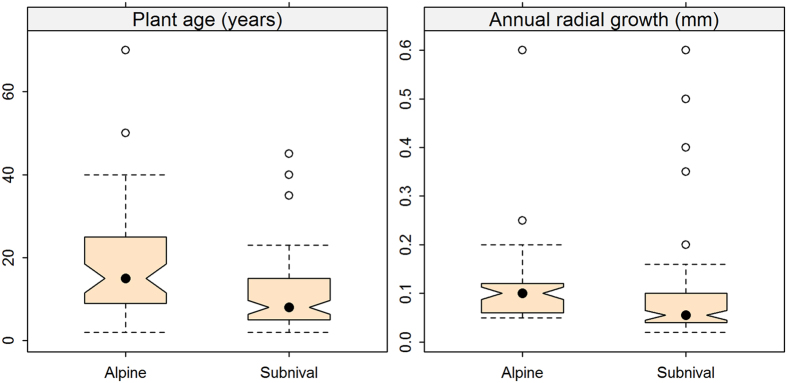
Difference in plant age and annual growth increment between alpine and subnival species. Non-overlapping notches in the boxes indicate the significance of between-group differences.

**Figure 5:**
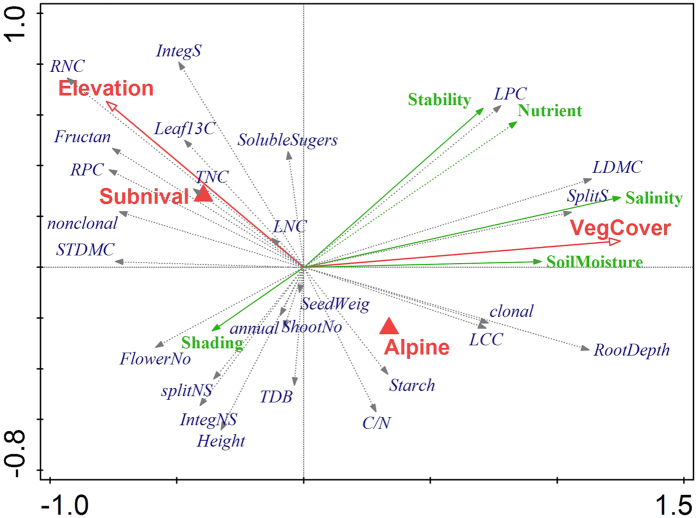
Redundancy analysis biplots (RDA) of community weighted mean values of plant functional traits (gray arrows) and habitat indicator values (green arrows) in relation to elevation, vegetation belt (alpine, subnival) and total vegetation cover (red color). The angles between arrows indicate correlations between variables (for explanation of trait and indicator values see Table 1). The environmental data explained 25.5% variability.

**Figure 6:**
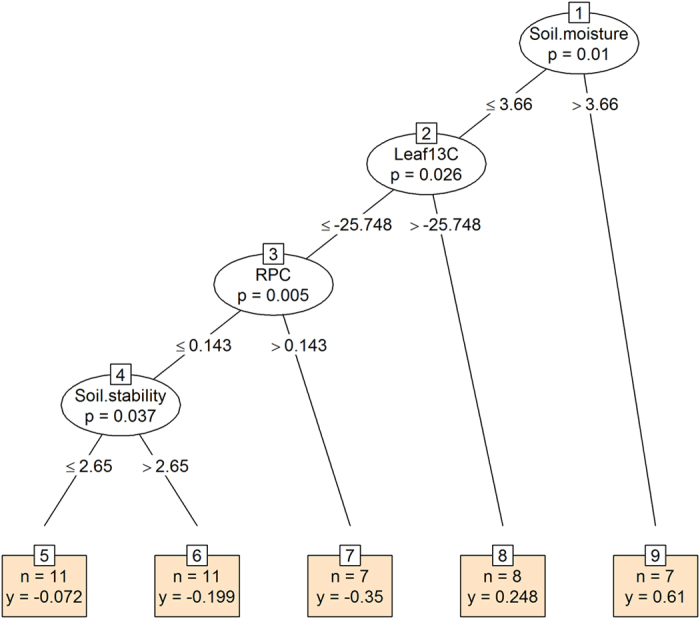
Life-history traits and ecological indicator values predicting species abundance changes in the alpine vegetation using the conditional inference tree. In each split of the tree, all species predictors are tested and the one that best discriminates between species is selected. The procedure goes on until no predictor significantly discriminates between species. The response variable (y) is the ordination score on the partial ordination first axis determined by a temporal change (boxes). Negative values indicate species declining over time, and positive values indicate species expanding. Each split of the tree is described by the trait or indicator value used at the split (ovals), the permutation-based significance of the split (*P*-value) (ovals) and the treshold values at which the split occurs (for explanation of trait and indicator values see Table 1). The number of species (*n*) is given at each terminal node (box).

Hence, the climate change in this arid region, involving warming and increasing precipitation, may not lead to the process of thermophilization as observed in cold and humid regions^3^, but rather to hygrophilization by increasing species from azonal wet habitats having otherwise small spatial extent in these arid mountains. We attribute the vegetation shift to the adverse effects of increasing daily maximum temperatures, which in combination with snowfall during growing season, creates freeze-thaw cycles. Unprecedented warming recorded in the period 2008–2012, with an increase in the mean daily maximum T by 2.4 °C and 1.4 °C per year for June and July, respectively, causes faster melting of snow instead of slow sublimation. The warming is accompanied by increasing diurnal fluctuations (Fig. 2C) as minimum daily T did not change significantly over time and hence the night frosts are still present. All this contributes to the recurrence of freeze-thaw actions (Supplementary Fig. S6). It is therefore the simultaneous increase in summer temperature and precipitation that can be detrimental for populations at the upper limit of plant distribution, in particular those with a single main root and adapted to dry and stable substrates. The abrupt damage to the alpine and subnival vegetation, which took place after snowfall summer 2010, will hardly be compensated soon because of the extremely slow growth in extreme elevations. Therefore, the impact of the increasing frequency of extreme precipitation events may, in the longer term, suppress the vegetation due to its immediate effect, while the positive effects of warming are relatively slow.

### Resilience to climate change

Dvorsky *et al.* 24 found that the generalist species with wide vertical ranges and optima in the alpine and steppe zone are more abundant in subnival flora of Ladakh than high-elevation specialists (see also Supplementary Figs S1 and S6). Subnival specialists with narrow elevational ranges represent 42% of the flora. Also among nine species forming uppermost populations above 6000 m are five generalists (*Aphragmus oxycarpus*, *Saussurea glacialis*, *Waldheimia tridactylites, Poa attenuata,* and *Draba altaica*) having optima at around 5100 m elevation and vertical ranges over 2000 meters long. The fact that subnival flora is composed of both specialist and generalist species suggests its relatively high resilience to climate change including both warming and increased frost disturbances in this region. Continued warming could potentially lead to thermophilization and increased abundance of steppic and alpine plants causing subnival specialists to decline at the alpine-nival ecotone. This was recently seen in wet lower mountains^5^,^35^ where subnival specialists already reached to mountain tops and hence cannot escape competition from alpine species by uphill migration. This is an unlikely scenerio in the arid NW Himalayas because the vast unglaciated areas between 6000–6400 m are available for range extension of the subnival species^24^,^42^,^45^. Complete extinction of subnival flora due to increasing precipitation and frost-disturbance is also unlikely given the high proportion of generalists among subnival taxa with their source populations at lower elevations capable of recolonizing sink habitats above.

## Conclusion

Knowledge of vegetation dynamics at the altitudinal limit of vascular plants is crucial for understanding plant migration processes, physiological limits and diversity changes^4^,^5^,^15^. Here, we explore the vegetation dynamics in the extreme altitudes of arid Ladakh, a rapidly warming region in the NW Himalayas, to test the ‘upward migration and thermophilization’ hypotheses, which predict an increase in absolute species distributional limits, vegetation cover and abundance of species adapted to warmer climate. We re-surveyed outpost populations after 10 years and permanent plots after 4 years from an area between 5500 and 6150 m.a.s.l. In addition, we determined the age and various functional and ecophysiological traits related to adaptations of plants to extreme altitude. We discovered several outpost populations during the re-exploration of the area above the upper vegetation limit determined 10 years before this study, supporting the upward migration hypothesis, with five subnival species extending their vertical range by 120–180 m, reaching an elevation of 6150 m. The plant age analysis corroborated the hypothesis of warming-driven uphill shift. However, the force of warming pushing plants upwards has been recently offset by extreme snowfall events as those in summer 2010. Vegetation resurvey of permanent plots revealed a substantial decrease in plant cover and compositional shift towards species preferring wetter habitats. Declining species have lower water-use efficiency, slow growth and main taproot, and are typical for the dry and cold NW Himalayas. Contrary, species whose cover increased were mostly clonal hygrophytes growing typically in wet soils. These vegetation changes are likely due to increasing soil moisture content and physical disturbance. Simultaneous increase in summer temperature and precipitation which causes rapid snow melt and, coupled with frequent night frosts, generates frost heaves detrimental to subnival plants. These relatively short-term disturbance episodes can have long-lasting effects because slow growth rate of high-altitude plants is unlikely to compensate for such severe disturbance. By combining vegetation resurvey with anatomical age determination, we conclude that climate change in the arid Himalayas produces a complex pattern of plant responses involving uphill migration due to warming, and vegetation decline due to increasing precipitation and soil disturbance. Actual effects of climate change in arid mountains are thus more complex than usually thought.

## Material and Methods

The study was conducted on the southwest spur of the Tibetan Plateau in Eastern Ladakh (Fig. 1A), Jammu and Kashmir State, India, on a high-altitude plateau 15 km East of Tso Moriri Lake (33.1 N, 78.2 E) at elevations ranging from 5500 to 6150 m, between Chalung, Shukule and Chamser Kangri Peaks. The region is arid (Leh: 115 mm/yr, 3514 m, ca. 170 km NW of the study region, Gar: 54 mm/yr, 4232 m, ca. 160 km SE of the study region), because most of the summer monsoon precipitation is blocked by the main Himalaya Range. In the foothill desert areas around 3000–4500 m elevation, annual rainfall does not even reach 100 mm; at elevations above 4500 m precipitation tends to increase. Precipitation falling in summer above 5000 m is mostly snow. Winter precipitation is rather erratic and the snow layer is usually thin^46^. Soils have a coarse-grained structure, with a high percentage of large gravel, low water and organic matter contents, high pH (7–8) and relatively high concentrations of total N and P^47^.

### *In situ* climate measurements

To gain detailed information about inter-annual climate variation in the region, we set up a network of automatic HOBO and TOMST^®^ TMS microclimatic stations recording air and soil temperature (T), air relative humidity (RH) and soil moisture at hourly intervals (Fig. 2). Microclimatic stations have been in place since August 2008 to September 2014 and were placed at ~100 m vertical intervals. The climate records show that the growing season length, defined here as the number of days where the soil temperature constantly exceeded 0°C (frost-free days), shortens by 9.6 days per 100 m (from 90 to 28 frost-free days), while the mean temperature of the growing season decreases on average by 0.39°C per 100 m elevation (from 5.2 to 2.8°C) along the studied vertical gradient (5500–6150 m) encompassing the alpine and subnival vegetation. Soil moisture content, averaged for the entire growing season, is generally low and varies from about 4 to 15%, with the highest values recorded at around 5700 m.

To monitor the local snow conditions, an automatic PlantCam^TM^ camera (www.wingscapes.com) was placed on a slope facing the permanent plots and climate loggers in the period 2011– 2012, taking a photo every day at 10 a.m. The close relationship between measured air relative humidity and local snow conditions allow us to calculate frequency of snow events over the whole measurement period (for details see Dvorsky *et al.*^24^). Continuous snow cover usually occurs from February until April but with great differences between years (Fig. 2D). There were 86 days with snow cover in a late winter period (March–May) in 2009, 92 in 2010, 54 in 2011, 41 in 2012, and 89 in 2013 (Supplementary Fig. S4). Temperature and relative humidity fluctuations were low in the April–May periods of 2010 and 2013 (Supplementary Fig. S3), which coincided with unusually deep snow covers, followed by a warm June and July. This led to high soil water content and increased the risk of frost heave (Supplementary Fig. S5), with mean daily minimum T ranging from −2.4 to −10.5 °C (absolute daily minimum T for June-July range from −4.3 to −18.1 °C), while the mean daily maxima reached +14.5 °C (Fig. 2B). The summer 2010 was snowiest in past eight years, there were 26 snowfall days in 2009 between June to August, 62 in 2010, 26 in 2011, 26 in 2012, and 27 in 2013.

### Assessment of upward migration and thermophilization

To explore the possible uphill migration over the last decade, we examined the elevational distribution of subnival plant species in the study region (Supplementary Fig. S1). We compared plant distribution data collected in 2001–2003^42^,^45^,^48^ with our recent distributional data collected in the same region in 2011–2014^24^,^49^. Depending on habitat diversity and species richness, individual localities were searched for about 30 minutes to one day. The elevation of individual floristic records was measured with an altimeter (Thommen, Switzerland) and Garmin GPS and stored in a floristic database of Ladakh^42^,^48^.

To explore possible thermophilization of species assemblages, we analyzed temporal changes in species composition, cover and richness in 80 permanent plots. These plots were established within the alpine (41 plots, 5500–5800 m) and subnival zone (39 plots, 5800–6030 m) in 2009, on a gentle west-facing slope (3–5 degrees of inclination) and similar exposition. Each plot was 1 m^2^ in size, and divided into one hundred 10 × 10 cm subplots with all species of vascular plants and their cover being recorded. In 2012, we resurveyed all these plots following the same sampling protocol after a period of rapid climate warming (Fig. 2). The subnival zone was distinguished by a floristically-based nivality index^24^, the absence of steppe species and by the presence of subnival specialists. Also, the plots with cover >50% were all recorded below 5800 m (in most cases contained clonal graminoids such as *Carex sagaensis* and *Kobresia pygmaea*), marking transition between the alpine and subnival zone^24^,^45^.

### Plant age and growth determination

To evaluate the age and growth of plants in high elevation communities, we measured 426 individual specimens of 67 herbaceous species collected in the alpine (43 species) and subnival belt (24 species) in August–September 2012–2014 (Fig. 1A). Due to dry soils in the study region perennial plants often have deep tap-roots^50^ and distinct annual ring increments. All monocot species (21%) were excluded because they are not suitable for herbochronological analysis. Three to four adult undamaged individuals of each species were excavated and the washed roots placed in plastic bags containing ethanol until further analysis. The samples were analyzed in the Swiss Federal Research Institute WSL, in Birmensdorf, Switzerland by a method developed by Gärtner and Schweingruber^51^. Transverse, tangential and radial sections were cut from all studied individuals using a sliding microtome. Microscopic images of optimal ring width sequences were made for counting of the consecutive number of rings (Fig. 1D), and measuring the width of annual increments^51^. Plant ages were determined by ring counting in the oldest sections in the transition between the hypocotyl and the primary root (root collar). The possibility of non-transparent rings due to inappreciable growth in extremely cold or snowy summer cannot be excluded, therefore the numbers of counted rings represent minimum plant ages.

### Plant functional traits

To assess the biological mechanisms indicative of thermofilization, we measured several morphological and ecophysiological traits (see Table 1 for abbreviations of the traits). These were: plant height, total dry biomass, number of flowers and shoots, seed weight, rooting depth, leaf dry matter content, leaf carbon, nitrogen and phosphorus concentrations, leaf δ ^13^C, root nitrogen and phosphorus concentrations and the content and composition of nonstructural carbohydrates. Starch and fructans were analyzed using colorimetric methods. Soluble sugars, i.e. raffinose family oligosaccharides, sugar alcohols and simple sugars were quantified using high-performance anion exchange chromatography with pulsed amperometric detection (for details see Chlumská *et al.*^52^ and Supplementary Fig. S9). Leaf δC^13^, which measures the ratio of ^13^C over ^12^C (%), is an integrated, long-term measure of the ratio between internal and ambient CO_2_ concentrations (Ci/Ca) that reflects the intrinsic water use efficiency (WUE) of plants^53^.

The traits were measured in a minimum of 10 individuals (up to 50 for some species) from various elevations within the studied region and the average values were calculated per species. In addition to quantitative traits, each species was classified as clonal or non-clonal based on the type of belowground organs (rhizomes with adventitious roots versus primary tap-roots), and further into four space occupancy strategies, based on the rate of lateral spread (spreading–more than 10 cm per year; non-spreading–less than 10 cm per year) and persistence of connections between ramets (splitters–plants producing adventitious roots with main root decaying; integrators–plants not producing adventitious roots and/or with perennial main root^50^).

### Habitat preference

In addition to plant traits, we included information on high-altitude species’ ecological optima in order to predict species’ response to climate change. Species’ optima on five environmental gradients were derived from vegetation composition of 369 plots (each 100 m^2^) sampled in a stratified design to cover major vegetation types over the study area^24^,^45^,^48^. On each sampled plot, five environmental variables were estimated: (1) soil/substrate stability: 1–unstable (screes, dunes, solifluction soils), 2–partly stable (grasslands, steppes), 3–stable (rocky crevices, *Kobresia pygmaea* mats); (2) light availability: 1–shaded (gorges, shaded rocky crevices, walls of stream banks), 2–partially shaded (dense vegetation cover), 3–full light (sparse vegetation cover); (3) soil moisture: 1–dry (substrate usually without visible traces of water), 2–mesic, 3–wet (water level regularly but transiently above soil surface), 4–permanent surface water; (4) soil fertility: 1–low (semi-deserts, steppes), 2–medium (alpine meadows), 3–high (stables, animal resting places); (5) soil salinity: 1–no salt deposits on soil surface, 2–salt deposits scarce, 3–salts forming a continuous crust. From these data, indicator values of a species were calculated as arithmetical means of indicator values in individual plots in which that species was recorded, weighed by the logarithm of its cover.

To obtain a robust estimate of the elevational optima of the species, we calculated response curves fitted with HOF models^54^. Species response curves were derived from 4,150 vegetation plots (each 100 m × 100 m) sampled over the entire Ladakh between 1999 and 2014 (Supplementary Figs S1 and S6). The dataset contain more than 122,000 records of occurrence of vascular plant species along exceptional elevational gradient from 2800 m to 6150 m^24^,^45^,^48^. Plant names follow Klimes and Dickore55.

### Physico-chemical characterization of the soil

To characterize physico-chemical parameters of soils, 50 g was taken as a composite of 5 subsamples from randomly selected points within each plot in 2009. The samples were air dried for 24 h on aluminum plate, after than placed into sterile polypropylene bags (Nasco Whirl-Pak^®^) and transported to the laboratory for analyses. In the laboratory, the soil samples were oven-dried at 100 °C, grinded in a mortar and sieved to 2 mm fraction after the removal of roots. Major cations (Ca^+2^ , Mg^+2^ , K^+^ and Na^+^) as well as nitrogen and phosphorus were measured in all soil samples. Cations were quantified through atomic absorption spectroscopy (AAS) using SpectrAA 640 (Varian Techtron) at the Analytical laboratory of Institute of Botany, Czech Republic. Ammonia, nitrate and total nitrogen were determined colourimetrically after Kjeldahl mineralization using automatic FIAstar 5010 Analyzer (Tecator). Phosphorus was determined colourimetrically after digestion in HClO 4 using SHIMADZU UV - 1650PC spectrophotometer. Other physico-chemical data were also measured: pH, water content, organic matter content (OM), and texture (fraction of particles >0.5 mm in diameter). The alpine soils had significantly higher total N, P, Ca concentrations and organic matter content and lower texture than subnival soils (Supplementary Fig. S12).

### Statistical analysis

To assess temporal changes in the plot-level vegetation records (number of vascular plant species, total plant cover, individual species’ frequency and cover) between the surveys (2009 vs 2012), and between the habitats (alpine vs subnival) and their interactions, we used generalized linear mixed-effect models (GLMM^56^,^57^). The tests were based on the restricted maximum likelihood (REML) approach approximated by chi-square distribution. The statistical significance was assessed by computing Bayesian highest probability (HPD) intervals using Markov chain Monte Carlo simulations (999 permutation in each test).

To explore shifts in species composition, we performed multivariate ordination analysis RDA, separately for the alpine and subnival belts. The variance partitioning procedure was performed in RDA with explanatory variables (year) and co-variables (elevation, plot identity) to remove their effects and to obtain a net effect of the year. To assess whether compositional change is driven by uniform shift among species or rather by differential species responses, RDA was performed both with and without standardization by sample norm^58^. Compositional differences in plant assemblages were tested by 999 permutations.

To explore further the ecological causes for the species temporal responses, we used plant functional traits and habitat indicator values in explaining the vegetation changes. Two approaches were employed: (1) *plot-based analysis using trait averages* (weighted by log-transformed species cover abundance; hereafter *CWM*, community weighted means), compared between two sampling periods using GLMM as described above. Because obtained results reflect mainly the features of dominant species, we also used an alternative approach which places individual taxa into focus instead of individual plots: (2) *species-based analysis of the trait-environment relation* was a two-step analysis, in which the temporal change of individual species was first quantified (by scores on the first ordination axis of partial RDA, with the year as the only explanatory variable and elevation and plot identity as covariables) and then those scores were related to the traits and habitat indicator values of individual species using a *conditional inference trees*^59^. Conditional inference trees has several advantages over other classification and regression trees algorithms, including the statistical testing of each split through permutation, no need for problematic pruning of over-fitted trees, and no selection bias towards variables with many possible splits or missing values.

## Additional Information

**How to cite this article**: Dolezal, J. *et al.* Vegetation dynamics at the upper elevational limit of vascular plants in Himalaya. *Sci. Rep.* **6**, 24881; doi: 10.1038/srep24881 (2016).

## Supplementary Material

Supplementary Information

## Figures and Tables

**Table 1:** Comparison of plant traits and indicator values between the alpine and subnival vegetation and the shift in trait community-weighted means between two sampling periods from 2009 to 2012.

Traits and habitat preferences	Abbreviation	Alpine09	Subnival09	P	Alpine_diff_2009–2012	P	Subnival_diff_2009–2012	P
Plant height (cm)	Height	14.00	11.00	↓**	0.204		1.058	↑*
Leaf nitrogen content (%)	LNC	2.26	2.42	↑*	−0.004		−0.007	
Leaf phosphorus content (%)	LPC	0.18	0.17		0.001		0.002	
Leaf carbon content (%)	LCC	42.84	41.59	↓***	0.169	↑***	0.414	↑*
Leaf carbon/nitrogen ratio	C:N	20.13	18.40	↓***	0.140		0.301	
Leaf δC^13^carbon (%)	C^13^	−26.21	−26.05		0.001		−0.008	
Leaf dry matter content (mg/g)	LDMC	176.63	131.84	↓**	7.140	↑*	1.608	
Stem dry matter content (mg/g)	STDMC	326.86	329.70		−14.39	↓**	52.84	↑a
Root nitrogen content (%)	RNC	1.16	1.52	↑***	−0.032	↓***	−0.056	↓**
Root phosphorus content (%)	RPC	0.10	0.13	↑***	−0.003	↓***	−0.005	↓a
Starch content (%)	Starch	2.48	2.00		0.020		−0.043	
Fructan content (%)	Fructan	2.72	4.78	↑***	−0.055		−0.492	↓*
Soluble carbohydrates (%)	SolubleSugar	4.02	4.38	↑*	−0.100		−0.047	
Total non-structural carbohydrates	TNC	9.19	11.17	↑**	−0.149		−0.581	
Seed weight (g)	SeedWeig	0.04	0.03		−0.003	↓*	0.001	
Root depth (cm)	RootD	14.18	8.94	↓***	0.439	↑*	−0.434	↓*
Number of aboveground tillers	ShootNo	18.02	15.83		−0.386		1.105	
Number of flowers	FlowerNo	7.36	7.24		−0.340		0.466	
Total dry biomass (g)	TDB	2.45	1.25	↓*	−0.018		−0.060	
Clonal plants with rhizomes (%)	Clonal	54	36	↓**	2.7	↑*	6.4	↑*
Non-clonal with tap-roots (%)	Nonclonal	46	64	↑**	−2.7	↓*	−6.4	↓*
Nonspreading integrators (%)	IntegNS	34	34		−0.6		−5.6	↓*
Nonspreading splitters (%)	SplitNS	34	31		0.8		5.5	↑a
Spreading splitters (%)	SplitS	20	5	↓**	1.9	↑a	0.9	
Spreading integrators (%)	IntegS	12	30	↑**	−0.022	↓*	−0.9	
Indicator value_moisture	Moisture	2.84	2.68	↓***	0.049	↑***	0.012	
Indicator value_stability	Stability	2.46	2.42		0.002		0.011	
Indicator value_salinity	Salinity	0.33	0.12	↓***	0.013	↑a	0.003	
Indicator value_nutrient	Nutrient	0.98	0.98		0.007		0.005	
Indicator value _shading	Shading	0.09	0.09		−0.003		−0.006	
Elevational minima	Altmin	5056.6	5311.1	↑***	8.050		−29.9	↓*
Elevational optima	Altopt	5246.2	5534.4	↑***	−1.033		−24.2	↓*
Elevational maxima	Altmax	5708.9	5912.5	↑***	−1.469		−14.2	↓a
Elevational range	Altrange	652.3	601.4	↓*	−9.520	↓a	15.7	

An upward or downward pointing arrow indicates an increase or decrease from the alpine to subnival, or temporal shift. Type I error estimates ^a^*P* < 0.1, *P < 0.05, **P < 0.01, ***P < 0.001.
