# Supplementary material for: Vegetation dynamics at the upper elevational limit of vascular plants in Himalaya
Source: Sci Rep. 2016 May 4;6:24881. doi: 10.1038/srep24881 (PMC4855180; doi:10.1038/srep24881)
Supplement: Supplementary Information [file srep24881-s1.doc]

**Supplementary information**

**Vegetation dynamics at the upper elevational limit of vascular plants in Himalaya**

Jiri Dolezal^1,2^, Miroslav Dvorsky^1^, Martin Kopecky^1^, Pierre Liancourt^1^, Inga Hiiesalu^1^, Martin Macek^1^, Jan Altman^1^, Zuzana Chlumska^2^, Klara Rehakova^1^, Katerina Capkova^1,2^, Jakub Borovec^3^, Ondrej Mudrak^1^, Jan Wild^1^, Fritz Schweingruber^4^

Supplementary Table S1. **Changes in temperature (daily max, mean, range T) and snowfall totals (number of days with snow cover) from January 2009 and September 2014 at 5900 m in Eastern Ladakh, NW Himalayas.** Linear regression parameters (slope and intercept) with explained variance R^2^ and corresponding type I error estimate *P* are given.

| **Mean daily maximum temperature R^2^ P** | | | |
| --- | --- | --- | --- |
| **JAN:** | y = -0.0521x + 86.689 | 0.006 | 0.888 |
| **FEB:** | y = -0.3825x + 751.82 | 0.537 | 0.098 |
| **MAR:** | y = -0.4119x + 814.58 | 0.404 | 0.175 |
| **APR:** | y = -0.0831x + 157.66 | 0.003 | 0.925 |
| **MAY:** | y = 0.655x - 1320.9 | 0.119 | 0.503 |
| **JUN:** | y = 2.4281x - 4881 | 0.848 | 0.009 |
| **JUL:** | y = 1.4269x - 2859.9 | 0.791 | 0.018 |
| **AUG:** | y = 0.9502x - 1901.3 | 0.642 | 0.030 |
| **SEP:** | y = 1.0213x - 2050.4 | 0.736 | 0.014 |
| **OCT:** | y = 1.2472x - 2512.2 | 0.445 | 0.148 |
| **NOV:** | y = 0.6029x - 1222 | 0.161 | 0.430 |
| **DEC:** | y = 0.4399x - 898.96 | 0.218 | 0.350 |
| ***Mean daily temperature*** | | | |
| **JAN:** | y = -0.4803x + 943.73 | 0.307 | 0.254 |
| **FEB:** | y = -0.3852x + 753.92 | 0.670 | 0.046 |
| **MAR:** | y = -0.1389x + 261.3 | 0.308 | 0.253 |
| **APR:** | y = -0.1452x + 278.15 | 0.041 | 0.699 |
| **MAY:** | y = 0.2697x - 551.29 | 0.068 | 0.618 |
| **JUN:** | y = 1.2272x - 2471.8 | 0.709 | 0.035 |
| **JUL:** | y = 0.5909x - 1186 | 0.821 | 0.013 |
| **AUG:** | y = 0.1172x - 233.65 | 0.184 | 0.337 |
| **SEP:** | y = 0.69x - 1390 | 0.795 | 0.007 |
| **OCT:** | y = 1.0713x - 2165 | 0.562 | 0.085 |
| **NOV:** | y = 0.2028x - 423.9 | 0.052 | 0.665 |
| **DEC:** | y = 0.0861x - 192.97 | 0.047 | 0.680 |
| ***Diurnal temperature range*** | | |  |
| **JAN:** | y = 0.7819x - 1565.2 | 0.512 | 0.109 |
| **FEB:** | y = 0.0896x - 174.15 | 0.128 | 0.486 |
| **MAR:** | y = -0.4835x + 980.46 | 0.255 | 0.307 |
| **APR:** | y = 0.2162x - 426.23 | 0.013 | 0.830 |
| **MAY:** | y = 0.8x - 1598.2 | 0.195 | 0.381 |
| **JUN:** | y = 2.1471x - 4307.1 | 0.858 | 0.007 |
| **JUL:** | y = 1.3286x - 2658.9 | 0.632 | 0.058 |
| **AUG:** | y = 1.487x - 2977.4 | 0.506 | 0.073 |
| **SEP:** | y = 0.5792x - 1154.5 | 0.615 | 0.036 |
| **OCT:** | y = 0.2194x - 429.53 | 0.066 | 0.624 |
| **NOV:** | y = 0.6566x - 1308.9 | 0.261 | 0.300 |
| **DEC:** | y = 0.5948x - 1186.3 | 0.393 | 0.183 |
| ***Snowfall total*** | | | |
| **JAN:** | y = -2.9535x + 5962 | 0.296 | 0.343 |
| **FEB:** | y = -0.5429x + 1116.8 | 0.035 | 0.722 |
| **MAR:** | y = 0.1714x - 316.16 | 0.006 | 0.882 |
| **APR:** | y = -2.8857x + 5827.8 | 0.286 | 0.274 |
| **MAY:** | y = -2.7714x + 5591.9 | 0.185 | 0.394 |
| **JUN:** | y = -4.6x + 9266.7 | 0.734 | 0.029 |
| **JUL:** | y = 1.1714x - 2348.8 | 0.185 | 0.394 |
| **AUG:** | y = -0.1786x + 369.68 | 0.005 | 0.887 |
| **SEP:** | y = -2.4643x + 4965.8 | 0.763 | 0.010 |
| **OCT:** | y = -3.6286x + 7309.1 | 0.312 | 0.249 |
| **NOV:** | y = -4.7674x + 9598.4 | 0.663 | 0.094 |
| **DEC:** | y = -2.2674x + 4574.5 | 0.250 | 0.391 |

Supplementary Table S2. **List of species recorded in 80 permanent plots 1 x 1 m.** New species found during the second survey are in bold. GLMM model, with plot identity as a random effect factor and year as fixed effect factor, was used to test for species-specific changes in cover abundance between the two sampling periods (all *P* values <0.1 indicate species cover decrease). Also shown are plant heights and elevational minima, maxima, optima and ranges.

| Species | **P** | **Plant height** | **Elevational distribution (m)** | | | |
| --- | --- | --- | --- | --- | --- | --- |
|  |  | (cm) | Min | Mean | Max | Range |
| *Actinocarya acaulis* | ns | 2.0 | 4615 | 5295 | 5625 | 1010 |
| *Aphragmus oxycarpus* | 0.025 | 4.3 | 3420 | 5334 | 6000 | 2580 |
| *Arenaria bryophylla* | 0.033 | 3.5 | 4105 | 5397 | 5910 | 1805 |
| *Artemisia minor* | ns | 11.0 | 4362 | 5069 | 5700 | 1339 |
| *Aster flaccidus* | ns | 10.0 | 3500 | 4667 | 5675 | 2175 |
| *Astragalus confertus* | ns | 13.0 | 4410 | 5422 | 5795 | 1395 |
| *Astragalus hendersonii* | ns | 5.9 | 4924 | 5342 | 5595 | 671 |
| *Astragalus strictus* | ns | 15.0 | 3145 | 4489 | 5525 | 2380 |
| *Carex borii* | ns | 5.8 | 4620 | 5306 | 5730 | 1110 |
| *Carex sagaensis* | 0.000 | 8.1 | 3600 | 5149 | 5800 | 2205 |
| ***Carex pseudofoetida*** |  | 12.0 | 3130 | 4855 | 5875 | 2745 |
| *Delphinium brunonianum* | ns | 31.6 | 3680 | 5079 | 5700 | 2020 |
| *Desideria pumila* | 0.000 | 2.0 | 5191 | 5813 | 5990 | 799 |
| *Draba altaica* | 0.000 | 2.5 | 3500 | 5409 | 6150 | 2500 |
| *Draba lasiophylla* | ns | 8.4 | 3330 | 5028 | 5725 | 2395 |
| *Draba oreades* | 0.032 | 1.9 | 4250 | 5616 | 6010 | 1760 |
| *Elymus schrenkianus* | ns | 35.0 | 3331 | 4536 | 5582 | 2251 |
| *Eritrichium hemisphaericum* | 0.000 | 1.7 | 5225 | 5942 | 5990 | 765 |
| *Festuca non-coelestis* | ns | 11.0 | 4775 | 5412 | 5738 | 963 |
| ***Halerpestes sarmentosa*** |  | 4.2 | 2680 | 2824 | 5485 | 2805 |
| ***Hedinia tibetica*** |  | 12.0 | 4575 | 5434 | 5815 | 1240 |
| *Kobresia pygmaea* | ns | 3.5 | 4000 | 5171 | 5766 | 1766 |
| *Kobresia schoenoides* | ns | 20.3 | 2680 | 4602 | 5650 | 2970 |
| ***Koenigia islandica*** |  | 10.0 | 2880 | 5007 | 5725 | 2845 |
| *Ladakiella klimesii* | 0.000 | 4.5 | 5350 | 5862 | 6150 | 660 |
| *Nepeta longibracteata* | ns | 4.2 | 4390 | 5316 | 5775 | 1485 |
| *Oxytropis chiliophylla* | 0.106 | 8.4 | 4640 | 5266 | 5795 | 1165 |
| *Oxytropis platysema* | 0.000 | 9.5 | 4260 | 5228 | 5795 | 1545 |
| *Pegaeophyton scapiflorum* | 0.014 | 2.8 | 4020 | 5567 | 5920 | 1900 |
| *Poa attenuata* | 0.000 | 35.0 | 3245 | 5007 | 6150 | 2755 |
| ***Potentilla gelida*** |  | 13.1 | 3325 | 4967 | 5625 | 2300 |
| *Potentilla pamirica* | 0.000 | 4.3 | 3839 | 5157 | 5885 | 2046 |
| *Primula macrophylla* | ns | 17.7 | 3965 | 5228 | 5785 | 1820 |
| *Saussurea bracteata* | ns | 3.4 | 4540 | 5213 | 5740 | 1200 |
| *Saussurea glacialis* | 0.005 | 4.5 | 4250 | 5542 | 6010 | 1760 |
| *Saussurea inversa* | 0.019 | 3.7 | 4660 | 5843 | 6150 | 1310 |
| *Saussurea hypsipeta* | 0.035 | 7.0 | 4870 | 5853 | 6060 | 1190 |
| *Saxifraga cernua* | 0.064 | 8.5 | 4350 | 5462 | 5885 | 1535 |
| *Saxifraga nanella* | 0.006 | 1.7 | 4970 | 5765 | 5995 | 1025 |
| *Stellaria brachypetala* | ns | 20.0 | 2898 | 4103 | 5420 | 2522 |
| *Stellaria decumbens* | 0.000 | 2.5 | 3410 | 5630 | 6010 | 2600 |
| *Stellaria depressa* | 0.057 | 17.5 | 3660 | 5101 | 5950 | 2290 |
| *Stipa subsessiliflora* | ns | 31.0 | 4038 | 4944 | 5690 | 1653 |
| *Thalictrum alpinum* | ns | 11.1 | 3745 | 4956 | 5675 | 1930 |
| *Thylacospermum caespitosum* | 0.023 | 30.0 | 4200 | 5461 | 5960 | 1760 |
| *Waldheimia tridactylites* | ns | 7.0 | 3615 | 5424 | 6150 | 2415 |

**
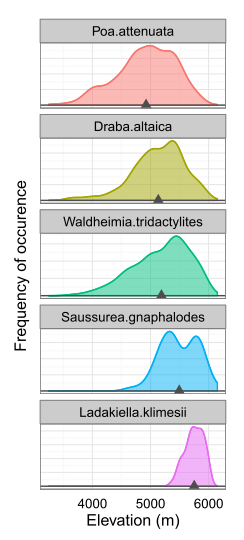

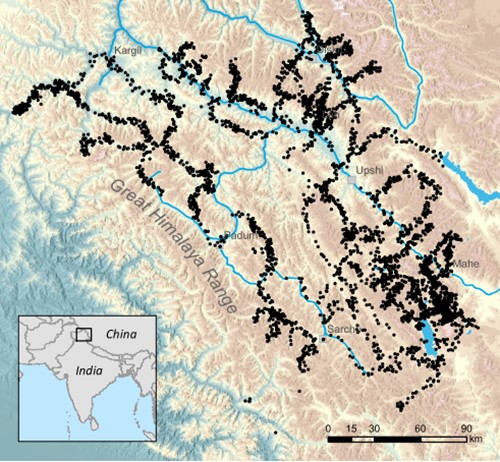
**

Supplementary Figure S1. **Study area in Ladakh, Indian NW Himalayas**. Floristic data were collected at 4,150 localities (black dots) spread over Ladakh area (to facilitate the visibility of overlapping plots, we randomly shifted plot coordinates up to 2 km). The map was created in software ArcGIS, ver. 10.1 (www.esri.com). The backround relief was derived from the SRTM data (http://www2.jpl.nasa.gov/srtm/, the version provided by http://srtm.csi.cgiar.org), and the river network was adapted from OpenStreetMap (www.openstreetmap.org).

Elevational ranges of five vascular plant species that significantly moved upwards in the past ten years in Eastern Ladakh near Tso Moriri Lake, where repeated monitoring of alpine and subnival vegetation was conducted. The curves represent the density distribution of sites with individual species presence and black triangles represent the mean elevation of these sites.


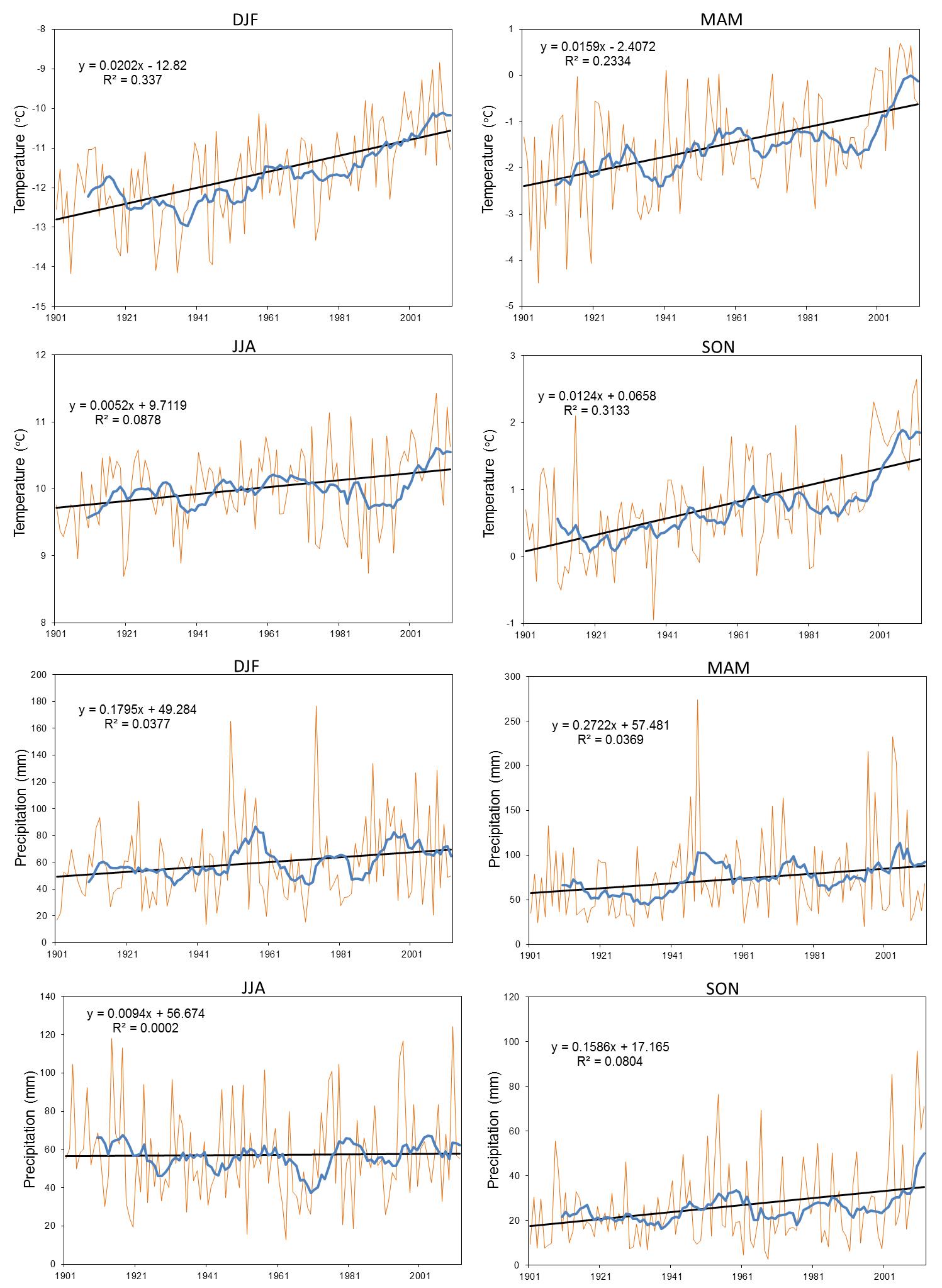


Supplementary Figure S2. **The long-term climatic changes in Ladakh.** The average temperature and precipitatin in the study area for the spring (MAM), summer (JJA), autumn (SON) and winter (DJF) periods, with smoothed values (10 year running mean) and fitted linear regression.The 0.5° x 0.5° gridded monthly climatic data from CRU TS 3.22 (Harris 2014) were used ([http://climexp.knmi.nl](http://climexp.knmi.nl/)). Data cover the period from 1901 – 2014.

Harris I, Jones PD, Osborn TJ, Lister DH (2014) Updated highresolution grids of monthly climatic observations—the CRU TS3.10 dataset. Int J Climatol 34:623–642.


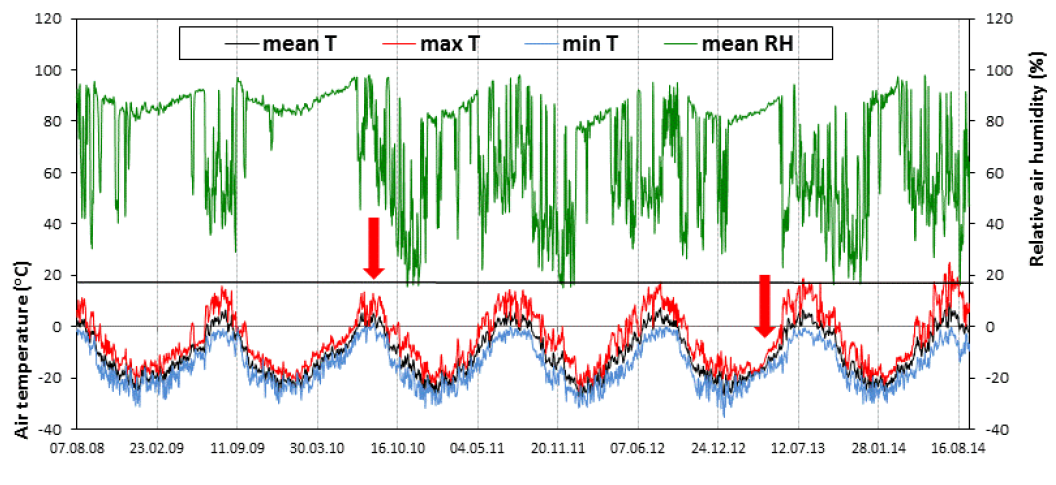


Supplementary Figure S3. **Climate at 5900 m.** The seven-year-long records of air temperature (T) and relative air humidity (RH), measured hourly 3 cm above the ground from August 2008 to September 2014, by a HOBO^TM^ U23 Pro v2 logger ([www.onsetcomp.com](http://www.onsetcomp.com/)).


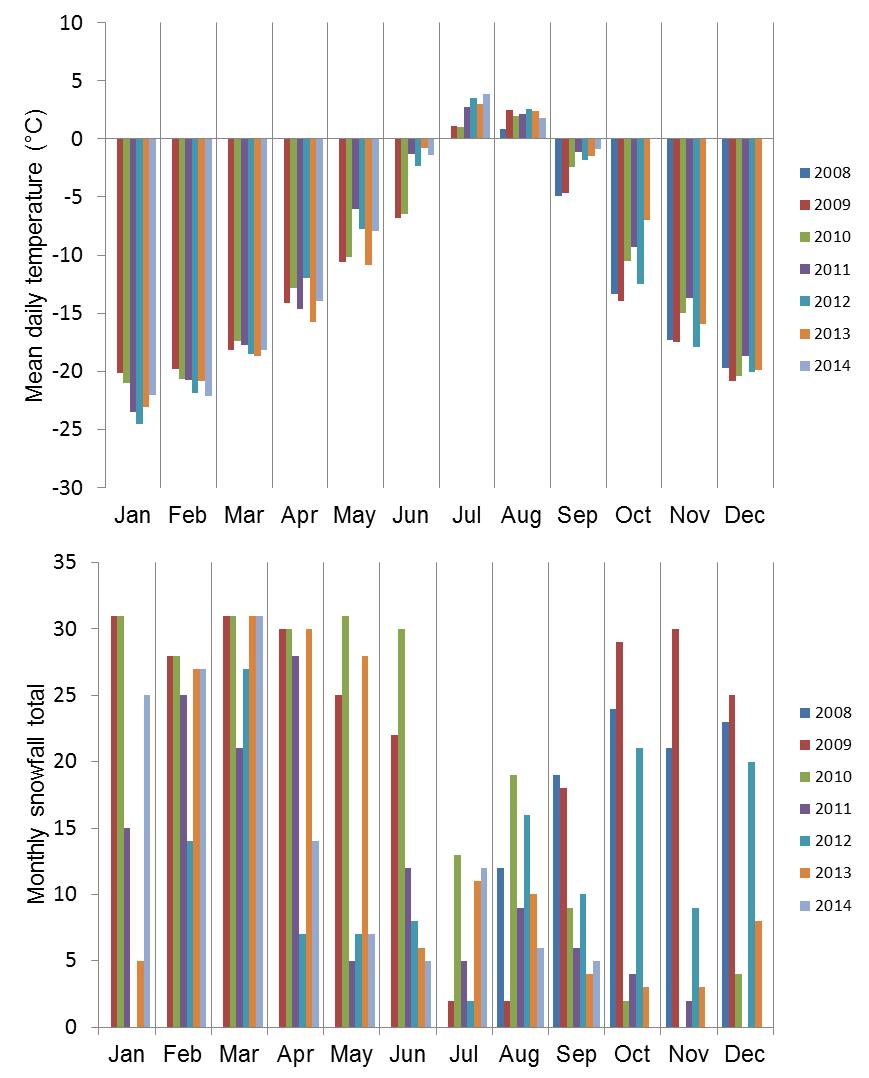


Supplementary Figure S4. **Climate at 5900 m.** Mean daily temperature and snowfall totals for individual months from August 2008 to September 2014 (see Table S2 for significant temporal trends).


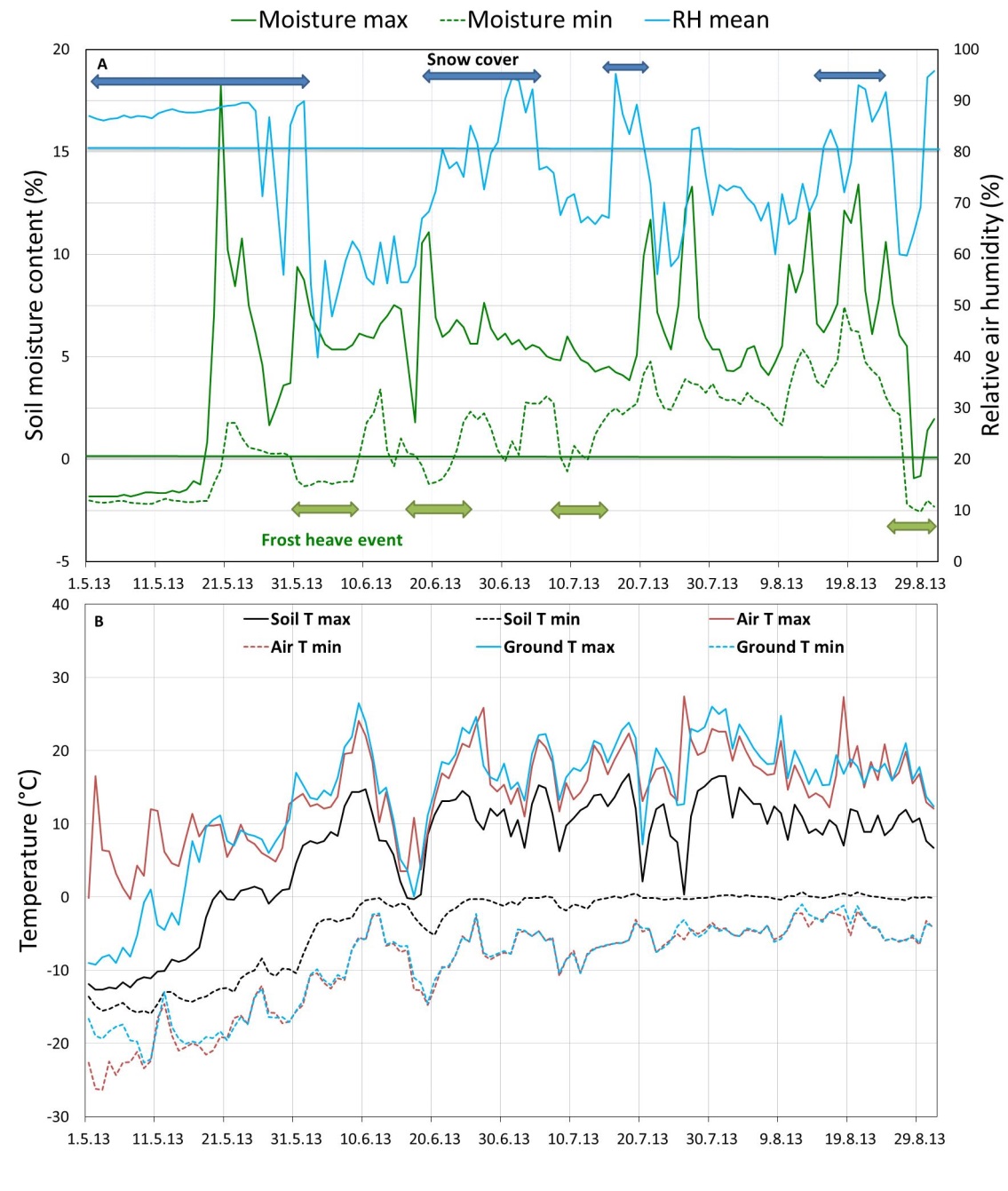


Supplementary Figure S5. **Example of frost heave events at 5900 m.** Continuous snow cover in 2013 lasted from February until May and was unusually deep, followed by a warm June and July. This increased the risk of frost heaving (soil water freezes and melts the same day) as an upwards swelling of soil during night freezing caused by an increasing presence of ice in wet soil after snowmelt. Days with snow cover/snowfall (blue arrows) were derived from relative air humidity (RH) measurements (mean daily values above 80%) verified by automatic PlantCam^TM^ camera snapshots. Snow temperature T (-8 cm) and soil moisture (0 to -10 cm), ground T and air T (+10 cm) were measured by TOMST logger.


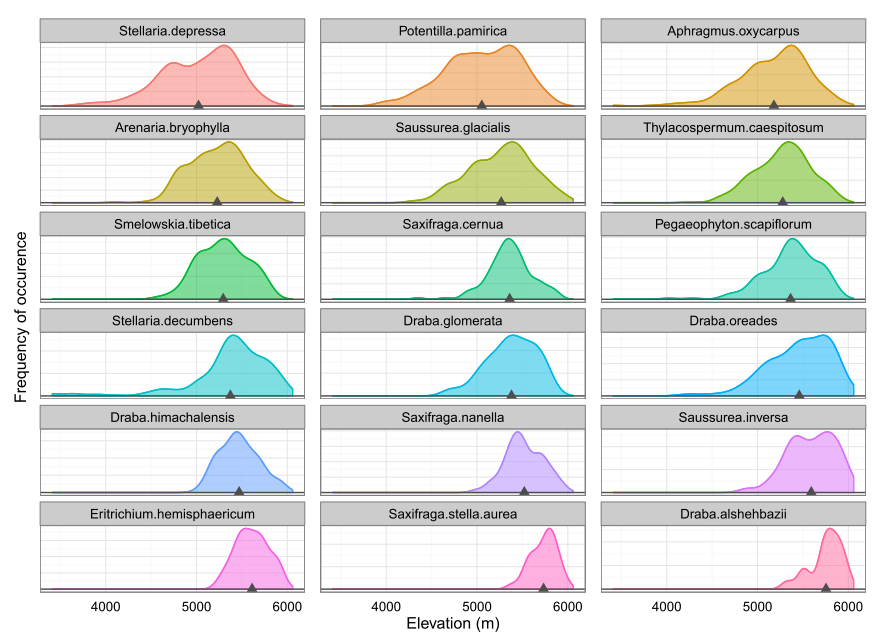


Supplementary Figure S6. **Species distribution along elevation gradient in Ladakh.** Only species found above 5800 m are shown. The curve represents the density distribution of sites with individual species presence and the black triangle represents the mean elevation of these sites. The subnival vegetation of Ladakh is not dominated by high-elevation specialists, but rather a mixture of species with heterogeneous distribution patterns. The prevailing are species centered at much lower elevations and having wide vertical ranges.


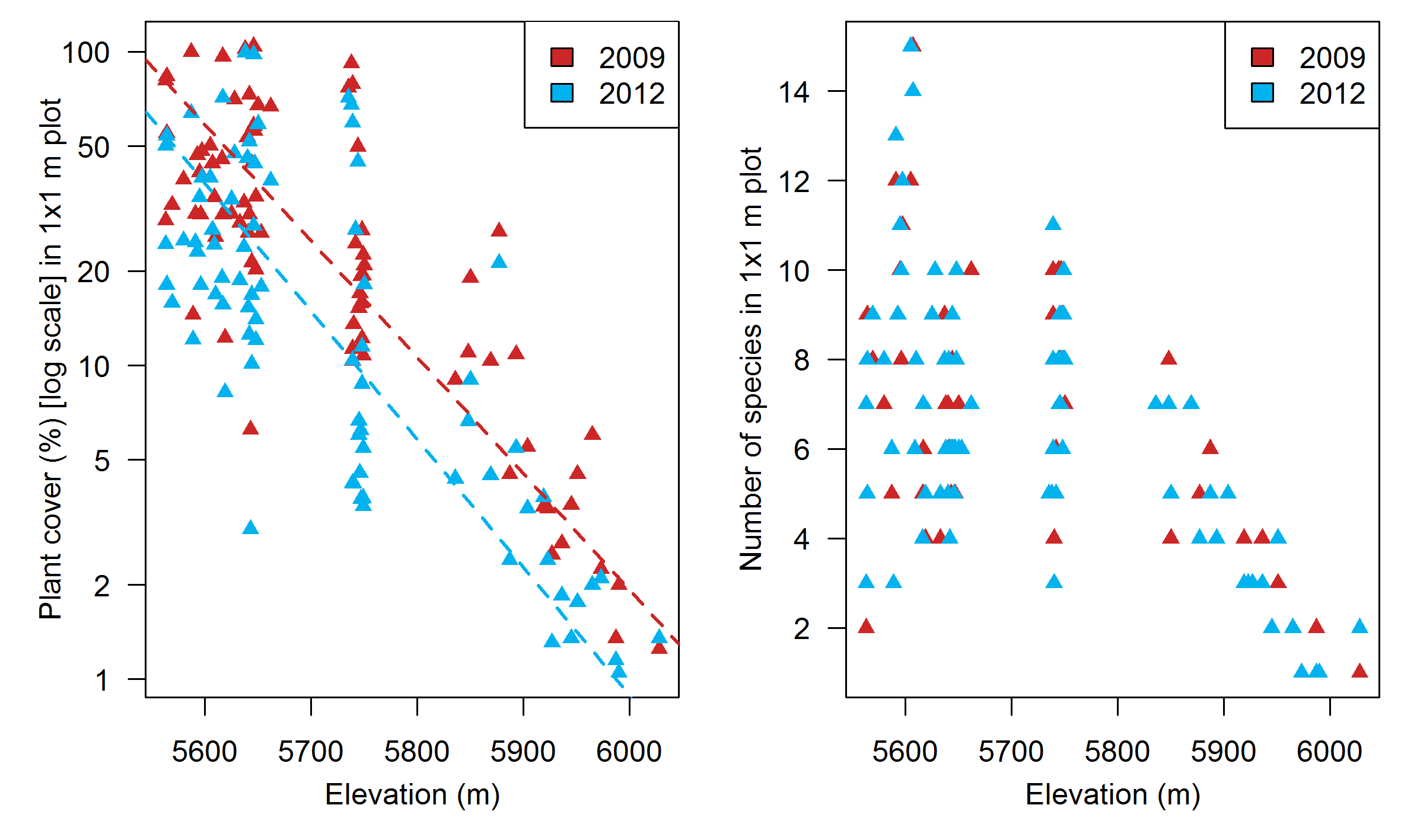


Supplementary Figure S7. **Changes in vegetation cover and number of species of subnival vegetation between 2009 and 2013 in 1 x 1 m plots.**


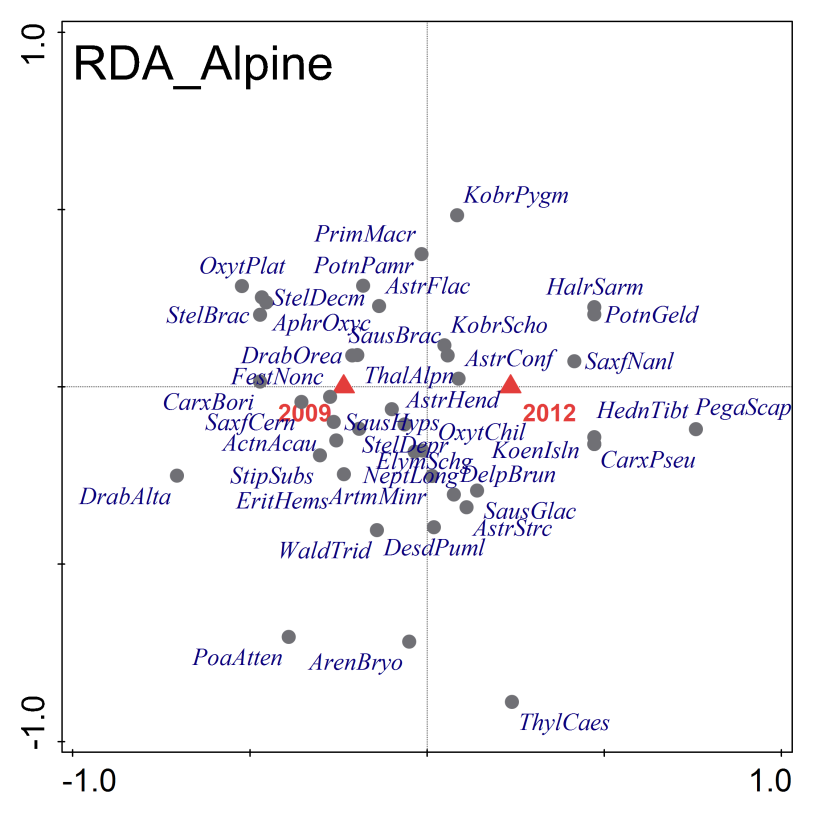

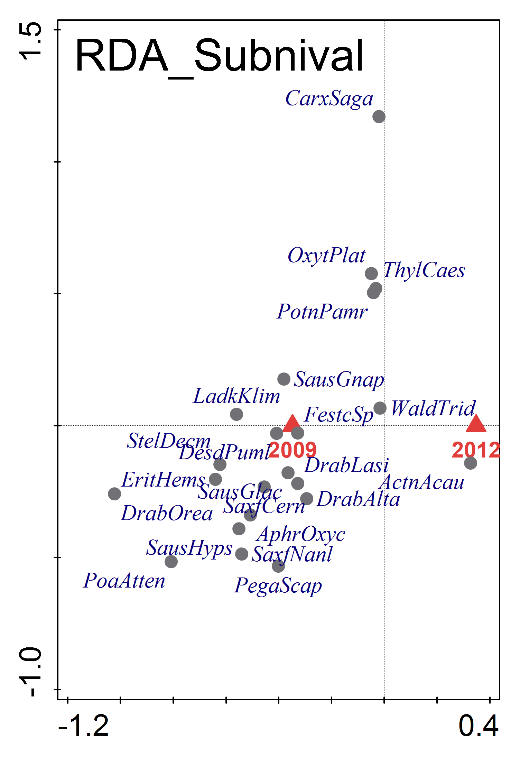


Supplementary Figure S8. **RDA ordination diagrams for compositional changes in alpine and subnival vegetation between 2009 and 2012.** The first four letters of generic and species names are given (see Supplementary Table S2 for full names).


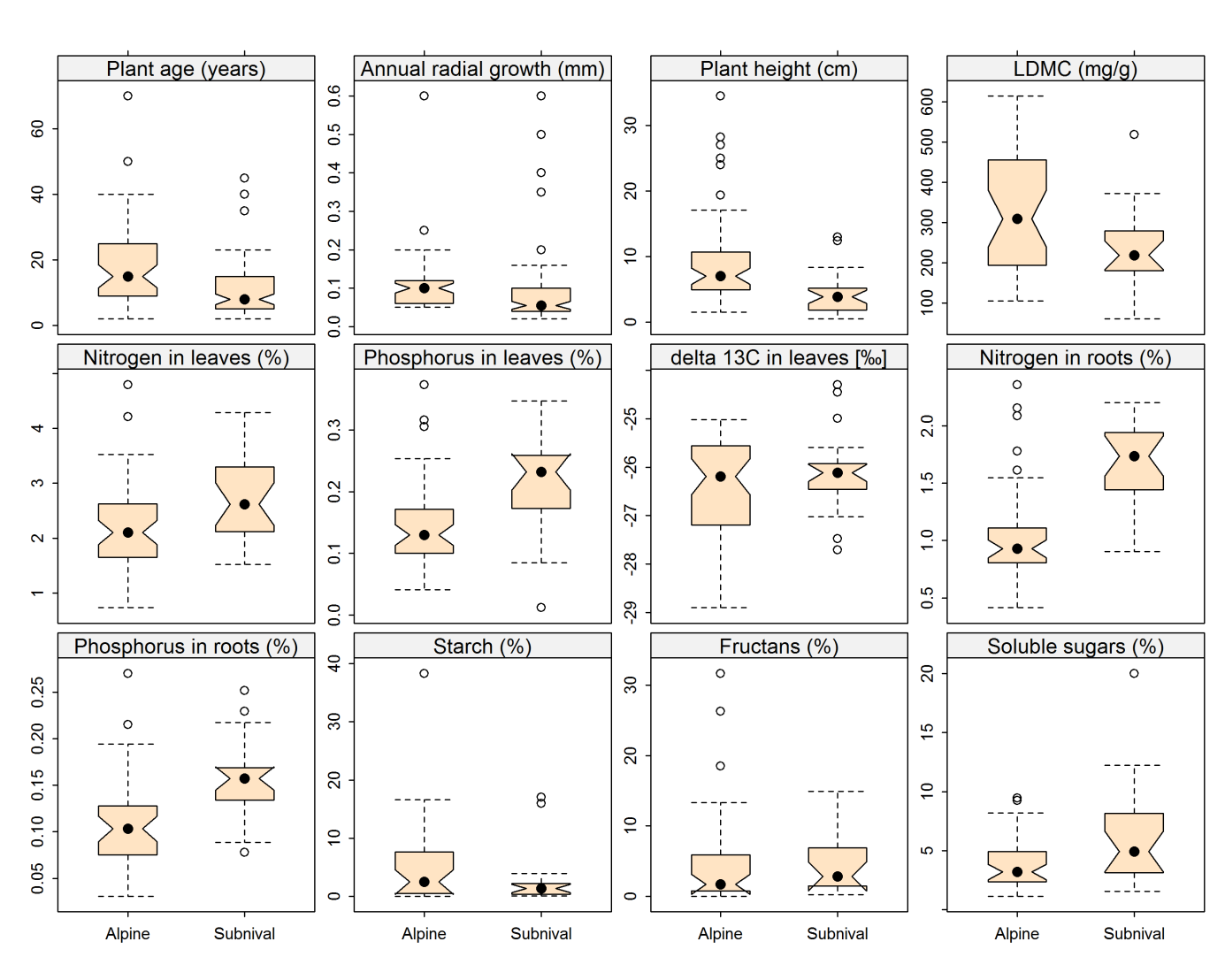


Supplementary Figure S9. **Comparison of plant life history traits between alpine (43 species) and subnival species (24 taxa above 5800 m) based on their mean values**. Soluble sugars include raffinose family oligosaccharides, sugar alcohols (glycerol, xylitol, arabitol, dulcitol, adonitol, myoInositol sorbitol, mannitol) and simple sugars (galactose, glucose, fructose, sucrose, D-galactosamine, rhamnose, arabinose, D-Glucosamine, xylose, mannose, D-allose, ribose, melibiose, glucoheptose, lactose, D-rafinose, D-talose, cellobiose, maltose), quantified using high-performance anion exchange chromatography with pulsed amperometric detection (for details see Chlumská et al. 2014). Boxes represent 25% – 75% of values, dots near the middle of the box are the medians, whiskers indicate the 1.5 interquartile range, and dots are outliers. Notches in the boxes indicate the significance of between-group differences: if notches of two groups do not overlap this is evidence that medians of these groups differ significantly. Notches that extend above the 75th percentile or below the 25th percentile indicate the high inter-sample variance and possible invalidity of the test that is based on the assumption of asymptotic normality of the median.


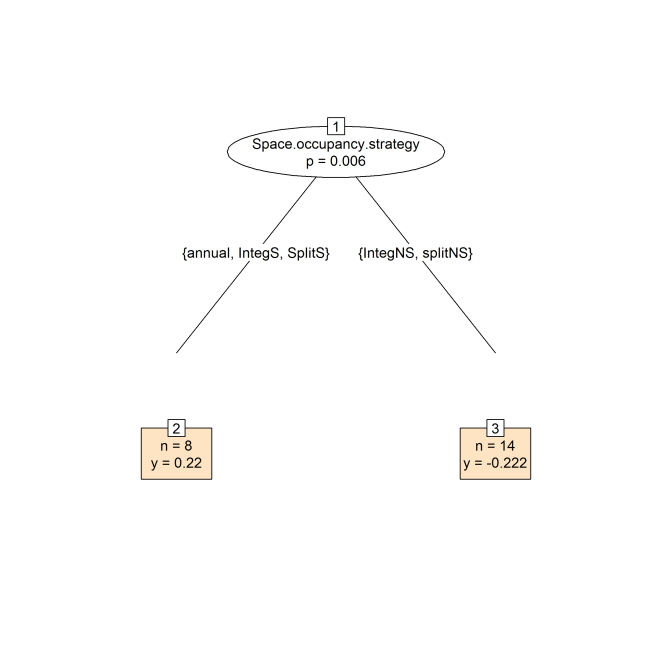


Supplementary Figure S10. **Life-history traits and ecological indicator values predicting species abundance changes in the subnival vegetation using a conditional inference tree.**


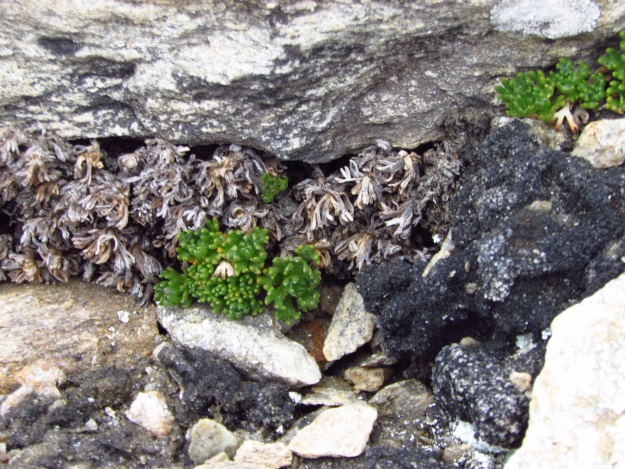

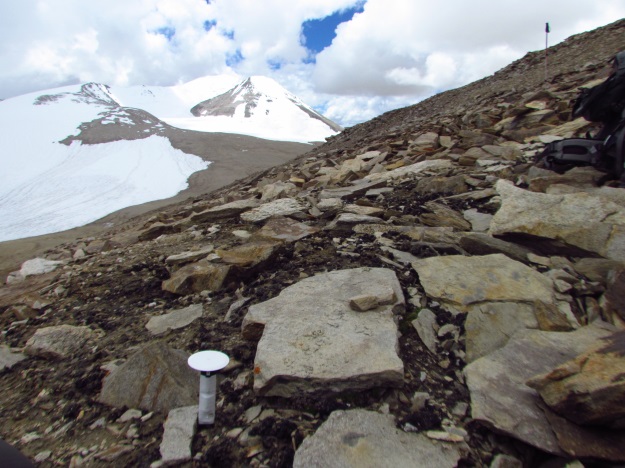


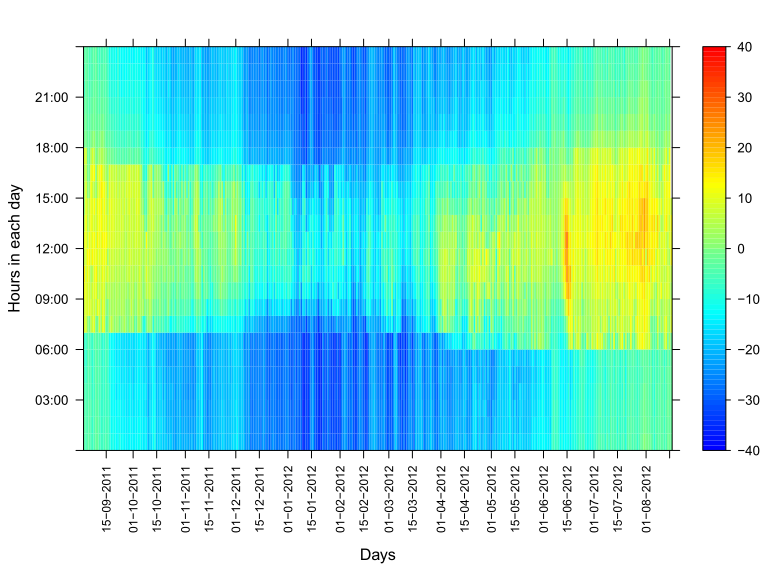


Supplementary Figure S11. **The highest and coldest site in the study area (6150 m) with vascular plants.** (Upper right) *Waldheimia tridactylites* population in the leeward side of a large boulder. The photographs was taken by the first author (J.D.) in August 2012. (Upper left) TOMST® TMS station recording air, surface and soil temperatures and soil moisture contents at 15 min intervals. Chamser Kangri peak (6660 m a.s.l.) in the background. (Bottom) Diurnal and seasonal course of air temperatures.


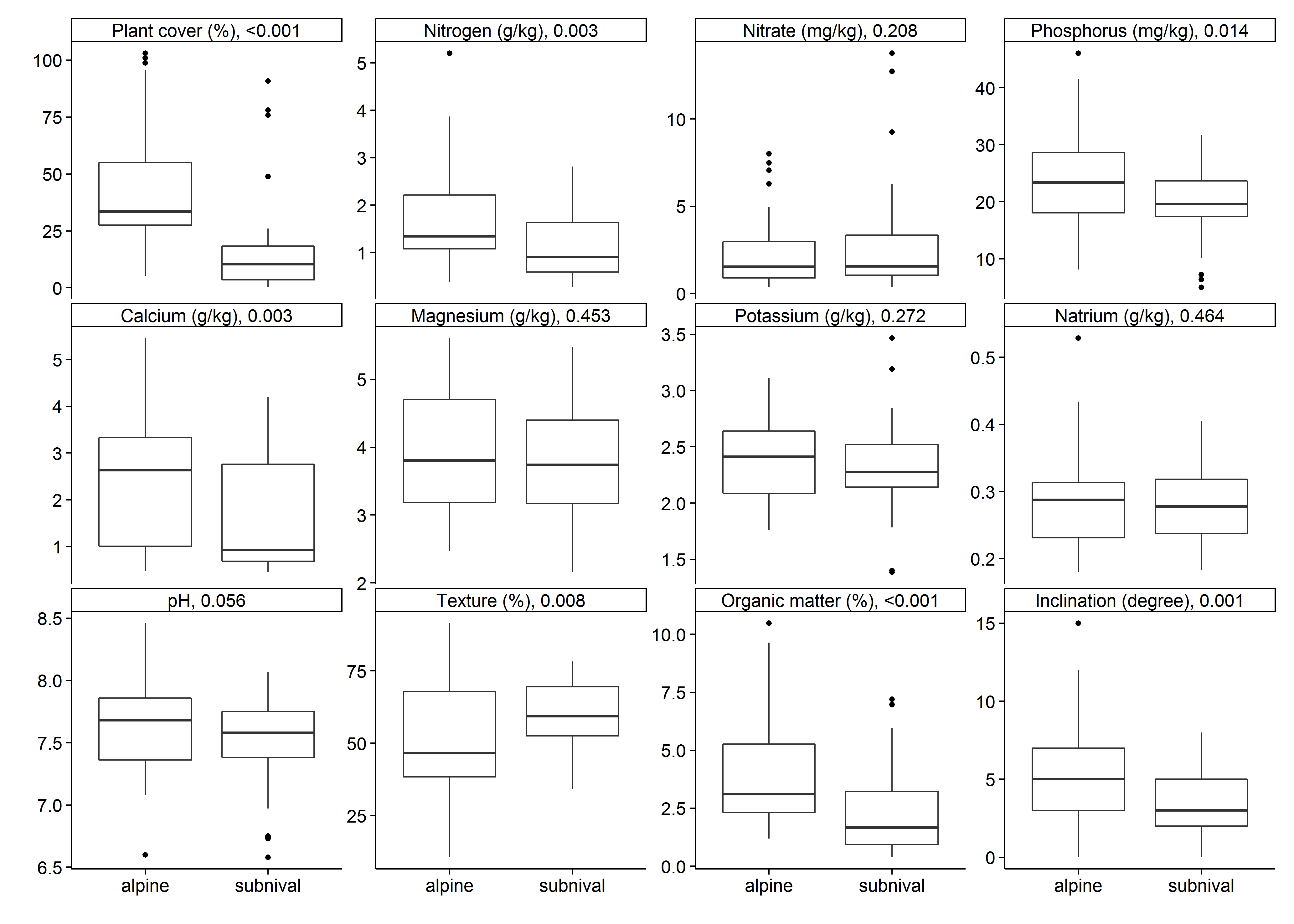


Supplementary Figure S12. **Plot-level comparison of soil chemismums, plant cover and slope.** Comparison of concentrations of total plant cover, soil nutrients, soil reaction (pH), soil texture (fraction of particles bigger than 0.5 mm in diameter), and slope inclination of permanent monitoring plots in the alpine and subnival vegetation on the western slope of Chamser Kangri, Tibetan Plateau. Generalized linear models were used to test for statistical differences between habitats (type I error estimate is given behind variable names).
